# Understory vegetation diversity patterns of *Platycladus orientalis* and *Pinus elliottii* communities in Central and Southern China

**DOI:** 10.1515/biol-2022-0791

**Published:** 2023-12-22

**Authors:** Nan Deng, Liu Caixia, Fengfeng Ma, Qingan Song, Yuxin Tian

**Affiliations:** Hunan Academy of Forestry, No. 658 Shaoshan Road, Changsha, 410004, Hunan, China; Hunan Cili Forest Ecosystem State Research Station, Cili, Changsha, 410004, Hunan, China

**Keywords:** afforestation, indicator species, species abundance distribution, species pool, understory vegetation diversity

## Abstract

As a vital component of arbor forests, understory vegetation serves as an essential buffer zone for storing carbon due to its strong capacity for community regeneration. This study aimed to identify the diversity pattern and construction mechanism of *Platycladus orientalis* and *Pinus elliottii* understory vegetation based on large-scale sample surveys. The Bayesian Information Criterion value of species abundance distribution (SAD) indicated that the Zipf and Zipf–Mandelbrot models were the best-fitting models. The SAD and gambin fitting results suggested that the *Pi. elliottii* community had a more balanced structure, with most species being relatively abundant. The multiple regression tree model detected four and six indicator species in *P. orientalis* and *Pi. elliottii* communities, respectively. The α-diversity index increased with a rise in altitude and showed a wavy curve with latitude. Linear regression between the β diversity and environmental and geographic distance indicated that the *P. orientalis* and *Pi. elliottii* understory communities tended to be dominated by different ecological processes. The partition of β diversity indicated that both communities were dominated by turnover processes, which were caused by environmental classification or spatial constraints. This study helped to understand the diversity maintenance in the *P. orientalis* and *Pi. elliottii* understory vegetation communities, and will benefit for diversity restoration and conservation of pure conifer forests.

## 1 Introduction

Unsustainable timber harvesting and deforestation have become a major global concern and reduced the forest ecosystems. The world’s forest area has been declining annually over the last 25 years [1,2]. The area of primitive forests has decreased sharply in many regions such as the Amazon Basin [3,4]. According to the UN report, a total of 420 million hectares of forests have been deforested globally since 1990, i.e., trees have been cut down and forest land converted to agriculture or infrastructure. In the last few decades, timber production and clear forest areas to be used for crops and grazing were the main objectives of forest management in China, where the forest cover is less than 13% before 1990. The multiple ecosystem services of forests have gained increasing attention, such as greenhouse gas emissions, and changes in the hydrological cycle [5–7] and protection of forests and afforestation are being carried out globally. China has undergone large-scale afforestation following prior deforestation. The planted forest area accounted for 8.0 × 10^7^ ha in 2018, and the forest coverage reached 23% in 2022. China has made remarkable achievements in forest restoration, and a quarter of the world’s new green space came from China between 2000 and 2017 [8]. The artificial forest has significant ecological benefits, but some ecological threats have emerged, such as biodiversity losses and changes to nutrient exchange, land degradation, invasion of alien species, and low forest quality [9–12]. Coniferous trees are widespread among afforestation species because of their tall and straight tree trunks. These trunks are versatile, serving as valuable resources for construction, vehicles, ships, and sleepers, as well as high-quality raw materials for papermaking and civil industry. Implementing the “ecological forests” policy has led to the protection of many areas to provide ecological services, constituting a crucial forest biodiversity repository. These protected forests rely on ecological succession to restore the structural and functional complexity, and allow natural disturbance dynamics, providing an example of passive rewilding [13]. Woody plant species encroachment does not universally degrade ecosystems [5,6,14,15] but rather expedites the recovery of understory shrub and herbaceous species and even imperiled avian species [16–18]. Understory vegetation is a vital component of forests and serves as an essential buffer for mitigating climate change due to its strong capacity for carbon accumulation [19,20]. The diversity pattern of understory vegetation not only reflects the structure and developmental stage but also reveals its dynamic adaptation to the environment [21].

Understanding biodiversity patterns and the mechanisms involved in their formation can contribute to sustainable and effective biodiversity conservation. The abundance of each species in an assemblage depends on several ecological characteristics [22]. The species abundance distribution (SAD) characterizes the proportional abundance of species in an ecological community and was proposed to be used to verify the mechanism underlying species assembly rules. The comparison of SAD models is often used to detect disturbance and damage to the ecosystem, explain resource allocation and interspecific associations among species, and so forth [16–18]. Biodiversity is usually divided into α and β diversities, with α diversity representing the number and evenness of species [23] and β diversity reflecting the species composition among communities on spatial or temporal scales [24]. α diversity is usually studied because it is easy to observe, and the spatial and temporal influences are ignored [25]. β diversity may reflect the dynamic of biodiversity patterns better than simple measures of α-diversity alone [26]. The niche and neutral theories are the two major theories that explain diversity gradients. Niche theory emphasizes the importance of the environment, and neutral theory holds that community dynamics is a random process. Moreover, dispersal limitations are considered to play an essential role in the community structure [27]. Comparing different aspects of diversity at different scales may be necessary to identify ecological processes [27].

The study areas were located in Central and Southern China, dominated by mountains and hills, forming a highly heterogeneous habitat unit and diversity pattern. The pure *Platycladus orientalis* and *Pinus elliottii* understory vegetation communities are the two typical afforestation species in the mountainous areas. However, the structure and underlying mechanisms governing the diversity of understory vegetation in these two forests remain unanswered. In this study, our objective was to (1) detect and compare the diversity patterns of the two understory vegetation communities, assessing the resilience of diversity structures and (2) reveal the community construction mechanism by β diversity to evaluate the further impacts of pure forest plantations on forest ecological succession.

## 2 Materials and methods

### 2.1 Data sources

**Figure 1:**
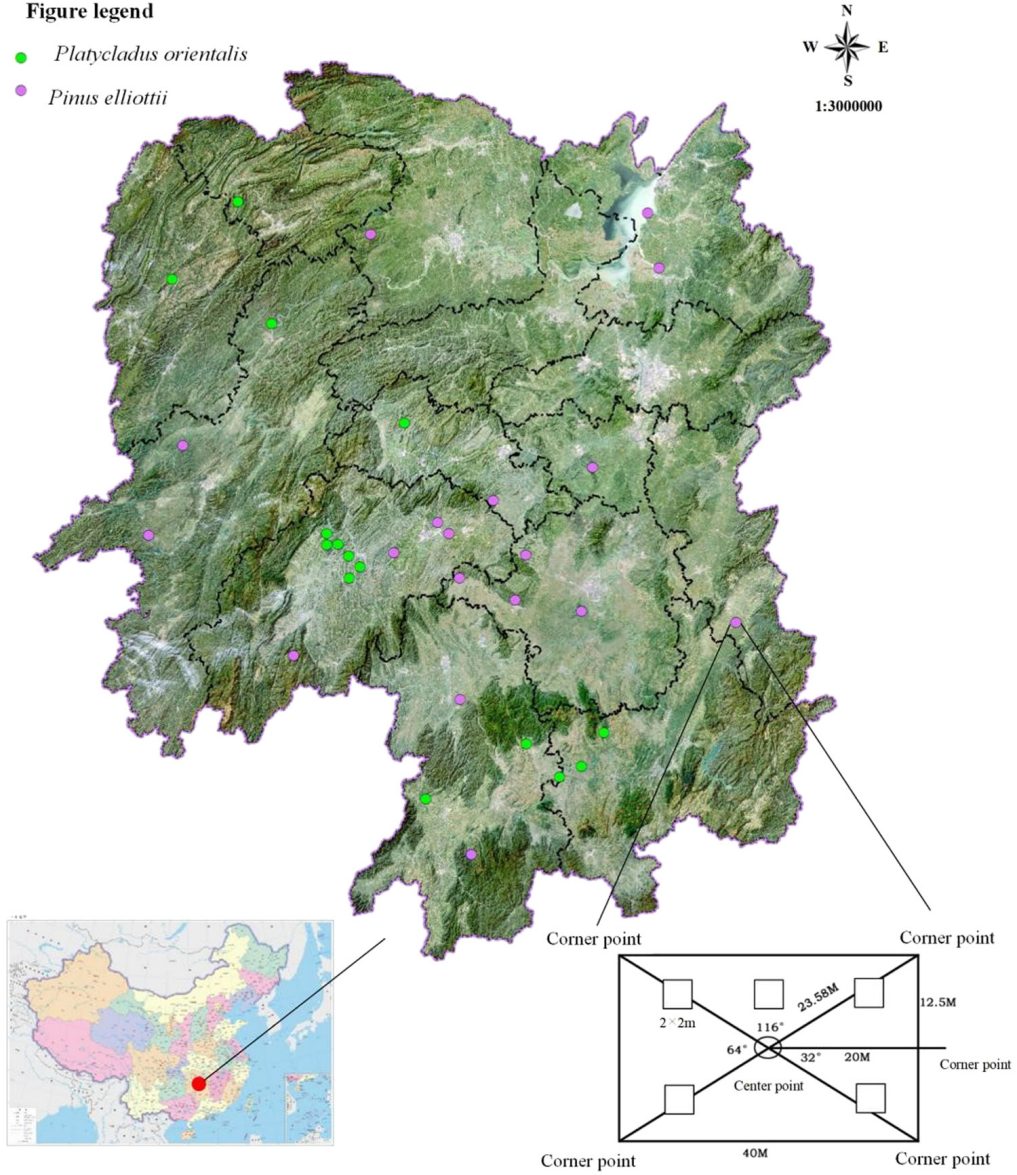
Study area and plot distribution.

The forest survey data were obtained from the forest fixed-area sample plot investigation database of Hunan ecological forests (updated in 2019), which contained 683 fixed-area sample plots distributed in subtropical areas. The plot size was 25 m (vertically to the contour line) × 40 m (parallel to the contour line). In each plot, trees with a DBH (diameter at breast height) exceeding 5 cm were measured. Additionally, five small shrub subplots (2 × 2 m^2^) were established within the plot to assess the characteristics of the understory plant community, plants below 5 m are recorded, each data of subplot are aggregated to represent the overall plot. Within these subplots, data including species composition and the number of shrubs were recorded. Stand dynamics factors, such as density canopy, slope position, and altitude for each plot, were also measured. For this study, the sites of pure *P. orientalis* (L.) Franco (15 plots) and *Pi. elliottii* Englem (18 plots) were selected from the database (Figure 1), with the majority of these plots representing plantation sites. Bioclimatic variables are considered as biologically meaningful variables, which are often used in species distribution modeling, and related ecological modeling techniques. For this study, 19 bioclimatic variables for each site were retrieved from the world climate database at the 30 s spatial resolution (WorldClim: http://www.worldclim.org/) (Table S1). The 19 bioclimatic and stand dynamics factors are selected as environment factors, which are used in multiple regression tree (MRT) and testing of β diversity.

### 2.2 Species diversity and fitting of SAD

The understory vegetation α diversity was assessed using the most commonly used diversity indices: richness, Shannon entropy, and Pielou’s evenness. SAD displayed logarithmic species abundances versus species rank to analyze types of abundance distributions. This study used five SAD modes, Brokenstick, Zipf, Log-normal, Niche preemption, and Zipf–Mandelbrot. The Zipf–Mandelbrot model fits the community structure through two parameters, beta and gamma. Gamma takes low values in highly organized systems with complex interactions among species, beta represents the potential diversity of the environment or niche diversification, taking higher values when the environment provides room for more alternatives [22]. The Akaike Information Criterion (AIC) and Bayesian Information Criterion (BIC) were used to evaluate models, and the Kolmogorov–Smirnov (K–S) test was used to test the models. The gambin model combined the γ distribution with a binomial sampling method, and a single free parameter (α) characterized the distribution shape [28]. We fitted the unimodal, bimodal, and trimodal versions of the gambin model to the diversity data, and then evaluated three models using the BIC. The analysis was conducted using the R package “vegan” and “sads.”

### 2.3 Species diversity and fitting of SAD

A regional species pool comprises all species available to colonize a focal site. Assessing variation in the size and composition of regional species pools is a way to include the potential influence of large-scale processes into analyses of community assembly [29]. Three models were used for predicting diversity potential in the study area: Chao [30,31], Jackknife, and Bootstrap [32]. The richness of each site was also estimated using the Chao and ACE models [30,31]. However, the probability of occurrence of each species in each site was calculated using the Beal smoothing model [33].

In the field of applied ecology, managers are often needed to predict plant communities by evaluating regional environmental types or finding species’ habitat preferences. The MRT model was used to explore the relationship between species composition and gradient of environmental factors though the R package “mvpart.” The indicator species analysis (ISA) is an effective method to determine the response of species to the environment, and is calculated according to the species distribution among groups. The indicator value indices of species were calculated, and the indicator value ranged from 0 to 1, with the higher value showing the better indicator [34]. The co-occurrence network was built to study the interactions between species based on the Jaccard similarity matrix, and different modules (species sets with high symbiotic frequency) and their internal associations were detected. The co-occurrence network analysis was conducted using the R package “igraph.”

### 2.4 Detection of driving factors in community construction

Dissimilarities among communities result from two different processes: turnover and nestedness [35,36]. In this study, dissimilarity coefficients were separated into turnover and nestedness components belonging to the Podani family [37]. In this study, the triple values of replacement, richness difference, and similarity corresponding to a specific point were graphically represented in a triangular graph. The envfit permutation function from the “vegan” package was used to detect significant affect factors. Subsequently, the trend curves of the selected factors were added to the canonical correspondence analysis (CCA) to analyze the species distribution pattern. The analysis helped assess important impact factors affecting the plant community.

## 3 Results

### 3.1 Fitting of SAD

**Figure 2:**
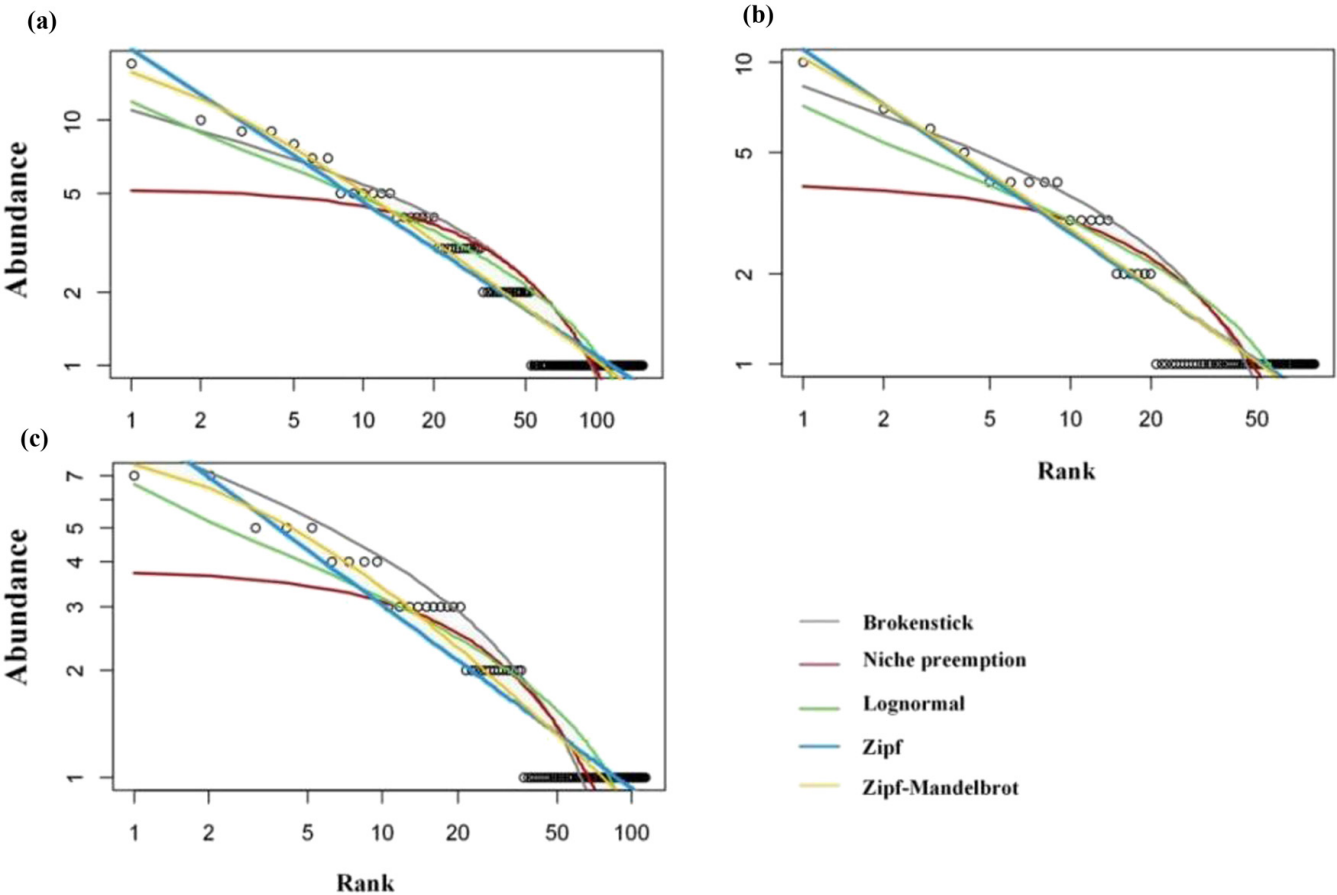
Fitting plots of different SAD models. (a)–(c) The whole, *P. orientalis*, and *Pi. elliottii* communities, respectively.

A total of 159 species were identified in two communities, with 82 in *P. orientalis* communities and 101 in *Pi. elliottii* communities. The altitude of the *P. orientalis* and *Pi. elliottii* plots ranged from 190 to 476 m and from 33 to 1,450 m, respectively, the stand density ranged from 280 to 3,350 and from 350 to 4,060/hm^2^, respectively. The SAD fitting results of the five models are shown in Figure 2 and Table 1. The K–S test results suggested that the *P* value of all SAD models were below 0.05, thereby proving that the rank abundance of the communities followed both the logseries and Log-normal-like-shaped distributions. The Zipf and Zipf–Mandelbrot models had the lowest AIC and BIC indexes, indicating the best-fitting effect. The parameter of Zipf–Mandelbrot was selected for SAD analysis due to the applicability of the model parameters. For parameter 2, the *P. orientalis* community had the highest value (0.64), followed by the *Pi. elliottii* community (0.69), and the whole community had the lowest value (0.72). The results indicated that the organizational structure of the *Pi. elliottii* community was more balanced than that of the *P. orientalis* community. For parameter 3, the *Pi. elliottii* community had the highest value (2.96), followed by the whole community (1.34), and the *P. orientalis* community had the lowest value (0.35). This suggested that the dominance of the dominant species of *Pi. elliottii* was stronger than that of the *P. orientalis* community.

**Table 1:** Fitting results of the five SAD models, parameters 1–3 represent c, gamma, and beta, respectively

Model	M1	M2	M3	M4	M5
**Type**	**Whole community**				
Parameter 1	—	0.016807	0.36805	0.063238	0.09347
Parameter 2	—	—	0.7712	−0.62369	−0.71619
Parameter 3	—	—	—	—	1.3424
Deviance	90.6565	62.2572	32.5307	10.0231	8.4317
AIC	465.0752	438.6759	410.9494	388.4418	388.8504
BIC	465.0752	441.7448	417.0872	394.5796	398.0571
*P* value of K–S test	1.06 × 10^−10^	1.02 × 10^−9^	1.09 × 10^−9^	4.64 × 10^−12^	4.64 × 10^−9^
**Type**	***P. orientalis*** **community**				
Parameter 1	—	0.028164	0.29719	0.079832	0.090943
Parameter 2	—	—	0.66489	−0.60374	−0.63913
Parameter 3	—	—	—	—	0.35302
Deviance	49.9958	28.0698	16.9759	5.4005	5.3006
AIC	236.0687	216.1428	207.0488	195.4734	197.3735
BIC	236.0687	218.5495	211.8622	200.2868	204.5937
*P* value of K–S test	8.36 × 10^−9^	2.32 × 10^−7^	1.71 × 10^−7^	1.95 × 10^−6^	4.18 × 10^−6^
**Type**	***Pi. elliottii*** **community**				
Parameter 1	—	0.02187	0.35288	0.057734	0.11386
Parameter 2	—	—	0.59455	−0.52523	−0.69361
Parameter 3	—	—	—	—	2.9623
Deviance	56.4835	20.4808	12.9996	6.3117	4.5878
AIC	290.1138	256.1111	250.6299	243.942	244.2181
BIC	290.1138	258.7262	255.8602	249.1723	252.0635
*P* value of K–S test	3.74 × 10^−8^	9.38 × 10^−7^	9.64 × 10^−8^	1.23 × 10^−9^	2.21 × 10^−6^

Additionally, the SAD was fitted by unimodal, bimodal, and trimodal gambin models. The unimodal gambin model was found to be the best-fitting model (Table 2). The parameter α was 0.43, 0.36, and 0.93 for the whole community, *P. orientalis* community, and *Pi. elliottii* community, respectively. A higher value of α indicated a strong restriction on diffusion, making it more likely for many rare species to disappear or become extinct within the community. The higher α index of *Pi. elliottii* indicated the Log-normal-like SADs in this community, suggesting a weak diffusion limit in *P. orientalis* community and that the individuals of the community are mostly immigrants.

**Table 2:** Fitting results of the gambin models

Models	Whole community	*P. orientalis* community	*Pi. elliottii* community
Unimodal	303.47	130.55	168.73
Trimodal	306.22	133.02	171.80
Bimodal	310.88	136.00	175.09
α value	0.4294	0.3555	0.9255

### 3.2 Co-occurrence network and indicator species

**Figure 3:**
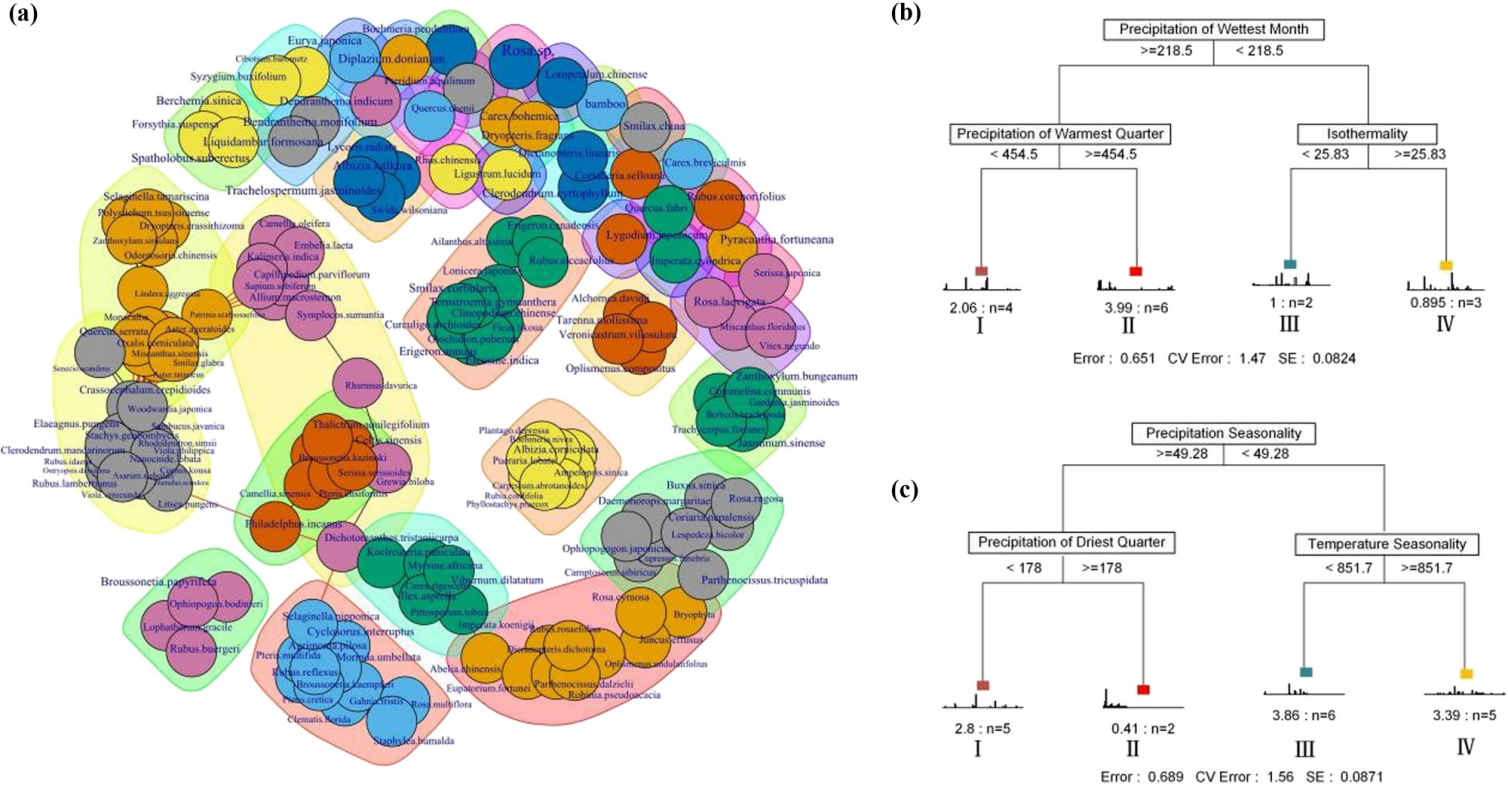
Co-occurrence network (a), and multiple regression tree for classifying *P. orientalis* (b)/*Pi. elliottii* (c). Different colors in the network represent different modules, the black line indicates the association within the module and the red line indicates the connection between the modules.

Three (Chao, Jackknife, and Bootstrap) models were used to estimate the richness potential of the two communities (Table 3). The richness predicted by the Chao model was the highest, followed by that predicted by the Jackknife model. The *Pi. elliottii* community had a bigger species pool. The probability of each species’ occurrence plot was also predicted using the Beal smoothing model (Figure S1). The co-occurrence network of the whole community is shown in Figure 3a. A total of 40 modules were detected; the modularity of the network was 0.81, indicating a close intermodule connection (Table 4).

**Table 3:** Prediction results of the species pool

Predicted species richness	*P. orientalis* community		*Pi. elliottii* community	
	Value	Variance	Value	Variance
Observed species richness	82	—	101	—
Chao model	381	144.79	257	57.66
Jackknife model	140	18.62	165	21.99
Bootstrap model	105	7.74	127	10.54

**Table 4:** Indicator species in each subgroup

Type	Species	Subgroup	Indicator value	*P* value
*P. orientalis*	*Rosa laevigata*	I	0.79	0.036
	*Serissa japonica*	IV	0.75	0.004
	*Juncus effusus*		1.0	0.002
	*Oplismenus undulatifolius*		0.67	0.036
*Pi. elliottii*	*Pteridium aquilinum*	I	0.82	0.001
	*Dryopteris fragrans*	II	0.94	0.013
	*Carex bohemica*		0.93	0.007
	*Symplocos sumuntia*		0.48	0.048
	*Ophiopogon bodinieri*		0.45	0.045
	*Loropetalum chinense*	III	0.64	0.042

Based on the results of the MRT model, the *P. orientalis* and *Pi. elliottii* communities were divided into four subgroups using three environmental factors (Figure 3b and c). The site of the *P. orientalis* community was divided by precipitation of the wettest month (threshold value = 218.5 mm), followed by precipitation of the warmest quarter (threshold value = 454.5 mm), and then isothermality (threshold value = 25.83). The site of the *Pi. elliottii* community was divided by precipitation seasonality (threshold value = 49.28 mm), followed by precipitation of the driest quarter (threshold value = 178 mm), and then temperature seasonality (threshold value = 851.7). In the subgroups, the bars in the chart represented the distribution frequency of the species, which served as an indicator of their sensitivity to the habitat gradient. The Monte Carlo test was used to test the significance (0.05) of each species. Four species were identified as indicator species in the *P. orientalis* community: *Rosa laevigata* was identified from subgroup I, and *Serissa japonica*, *Juncus effusus*, and *Oplismenus undulatifolius* from subgroup IV. Six species were identified as indicator species in the *Pi. elliottii* community: *Pteridium aquilinum* was identified from subgroup I; *Dryopteris fragrans*, *Carex bohemica*, *Symplocos sumuntia*, and *Ophiopogon bodinieri* from subgroup II; and *Loropetalum chinense* from subgroup III (Table 5).

**Table 5:** Envift permutation function of the whole plant community and environmental factors

Factor	CCA1	CCA2	*r* ^2^	*P* (>*r*)
Stand density	0.90814	−0.41866	0.0893	0.259
Altitude	−0.87534	−0.4835	0.1585	0.117
Tree coverage	0.20138	−0.97951	0.2734	**0.014**
Bio 1	0.98241	0.18671	0.2144	0.074
Bio 2	0.9496	−0.31346	0.0655	0.408
Bio 3	0.39917	−0.91688	0.1192	0.174
Bio 4	0.40891	0.91258	0.1292	0.16
Bio 5	0.92058	0.39056	0.1343	0.146
Bio 6	0.99812	−0.06129	0.2137	0.059
Bio 7	0.57383	0.81898	0.0858	0.301
Bio 8	0.9664	0.25703	0.3993	**0.007**
Bio 9	0.90533	−0.42471	0.0587	0.487
Bio 10	0.90813	0.41869	0.1784	0.095
Bio 11	0.99999	−0.00333	0.273	**0.029**
Bio 12	−0.02045	−0.99979	0.0704	0.364
Bio 13	0.47377	−0.88065	0.1411	0.128
Bio 14	−0.88446	−0.46661	0.0505	0.505
Bio 15	0.93792	−0.34685	0.0964	0.252
Bio 16	0.41508	−0.90978	0.1336	0.128
Bio 17	−0.96026	−0.2791	0.0987	0.256
Bio 18	0.83912	−0.54395	0.0224	0.731
Bio 19	−0.76197	−0.64761	0.0663	0.404

The bold values indicated the significant of 0.05%.

### 3.3 Distribution pattern of α and β diversities

**Figure 4:**
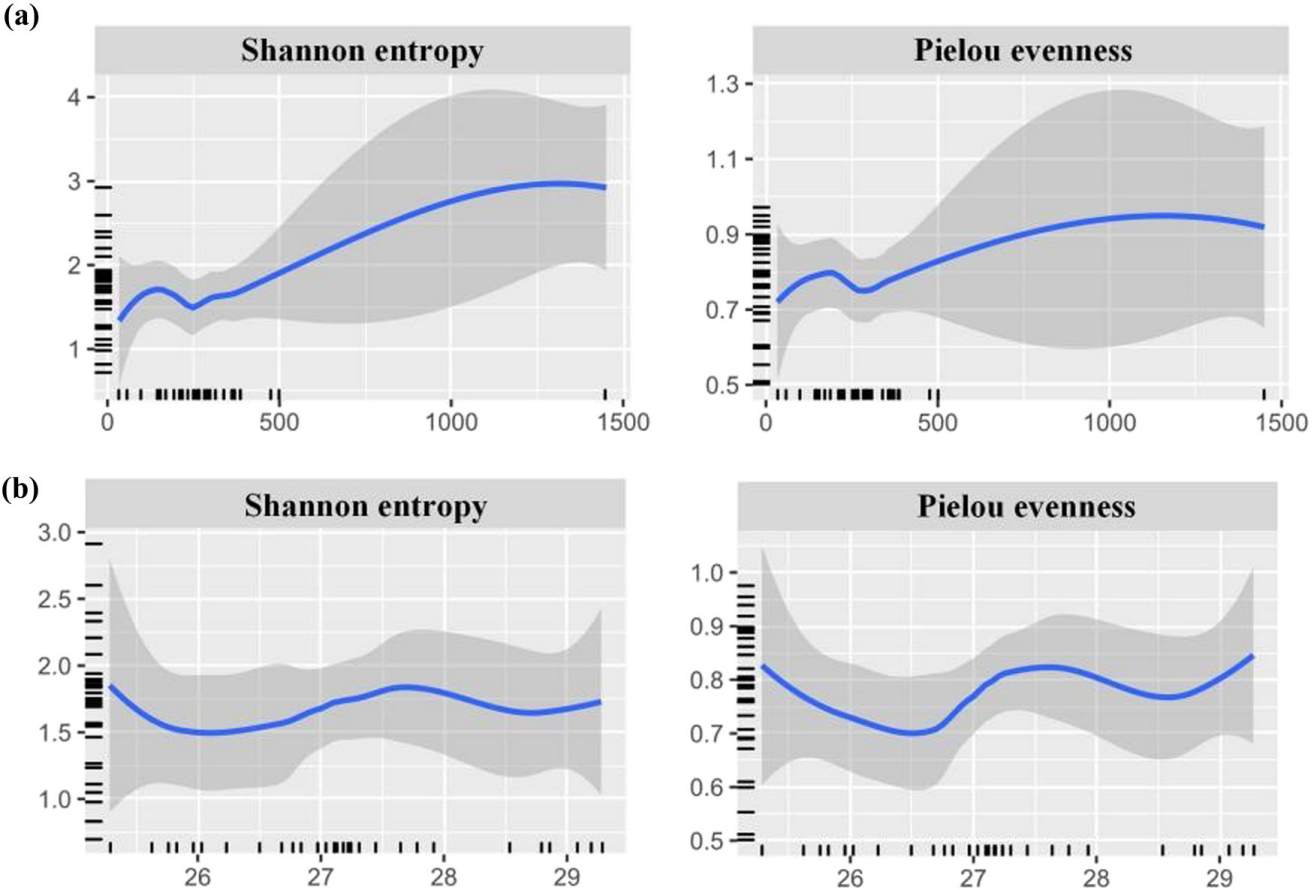
Variation in the diversity index along the altitude (a) and latitude (b) gradients.

The regression between the two diversity indexes of the whole community and altitude is presented in Figure 4a. The diversity index increased with a rise in the altitude, and then decreased. The regression between the diversity index and the latitude showed a wavy curve (Figure 4b).

**Figure 5:**
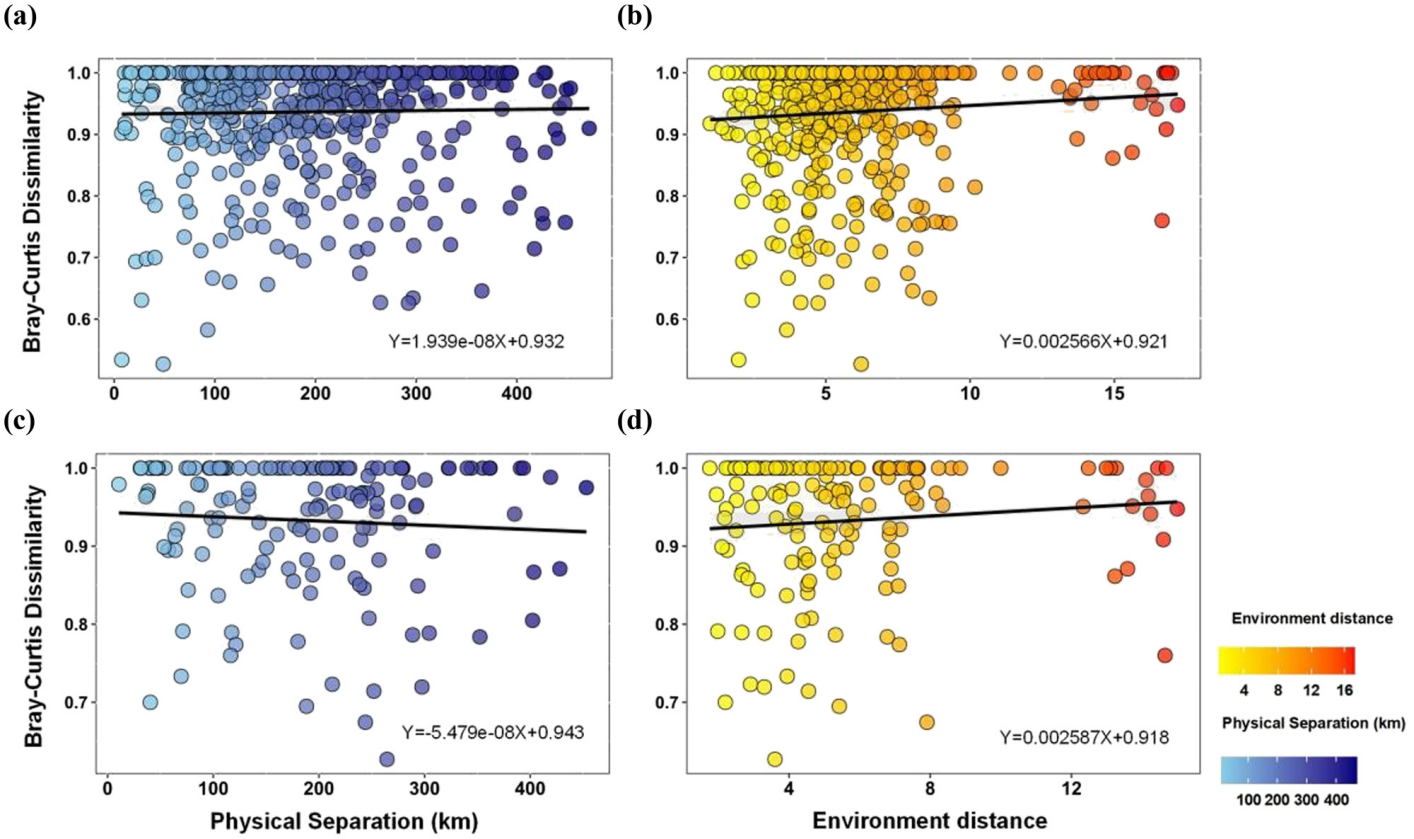
Variation in β diversity with environmental (yellow)/geographic distance (blue). (a) and (b) *P. orientalis* community; (c) and (d) *Pi. elliottii* community.

The linear regression between the β diversity and environmental/geographic distance showed that the species composition of the *P. orientalis* community showed a positive correlation with the environmental/geographic distance, indicating that both neutral effect and habitat filtering influenced the community establishment. The species composition of *Pi. elliottii* showed a negative correlation with the geographic distance and a positive correlation with the environmental distance, indicating that habitat filtering affected the community establishment (Figure 5).

**Figure 6:**
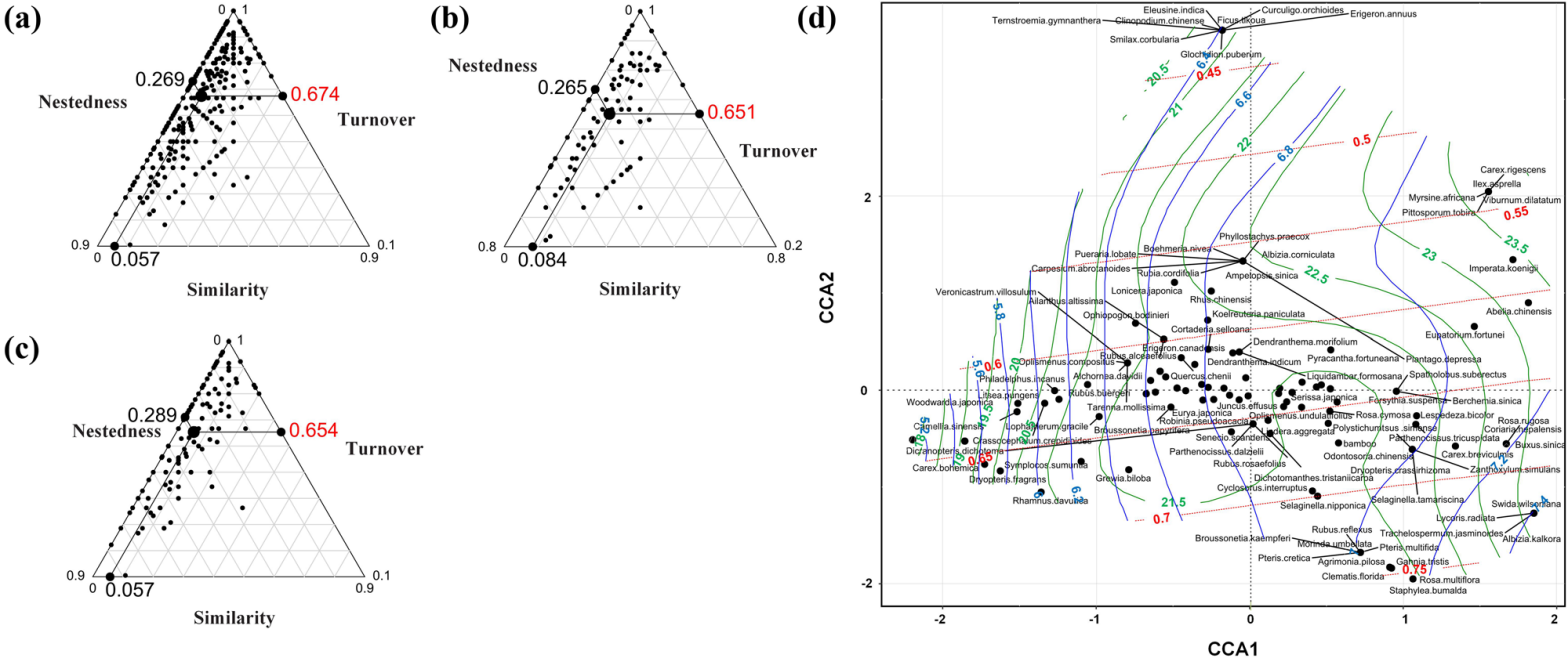
Triangular plots of the relationships among the pairs of plots and CCA analysis. (a–c) Triangular plots of the whole, *P. orientalis*, and *Pi. elliottii* communities, respectively. The large central dot in triangular plots is the centroid of the points, and the smaller dots represent the mean values of the similarity, turnover, and nestedness components. (d) CCA analysis of the whole community; red, green, and blue lines represent the trend curve of tree coverage, mean temperature of the wettest quarter, and mean temperature of the coldest quarter, respectively.

The β diversity was further partitioned into total replacement diversity and total richness difference diversity. The results indicated that the turnover processes dominated the dissimilarity of these community compositions. The turnover processes accounted for 33.7, 29.8, and 29.2% (community) of β diversity in the whole, *P. orientalis*, and *Pi. elliottii* communities, respectively. The nestedness had a low proportion of 13.5, 18.4, and 18.9% in the whole, *P. orientalis*, and *Pi. elliottii* communities, respectively. In triangular plots (Figure 6a–c), each point represented a pair of plots. Its position was determined by a triplet of values from the similarity, turnover, and nestedness matrixes. The graphs in the figure show that most of the points were distributed along the left edge. Additionally, the mean points along the turnover axis were more than 0.5 (0.674, 0.651, and 0.654). This indicated that the among-site variation was dominated by turnover, confirming the aforementioned values.

The assessment of environmental factors on the whole plant community (Table 6) revealed that the tree coverage (*r* ^2^ = 0.27, *P* < 0.05), mean temperature of the wettest quarter (Bio8, *i* ^2^ = 0.40, *P* < 0.05), and mean temperature of the coldest quarter (Bio11, *r* ^2^ = 0.27, *P* < 0.05) were the important factors affecting the distribution pattern of plants. Then, the trend curves of the three factors were added into the CCA analysis (Figure 6d). These species had significant distribution patterns; some species, such as *Ternstroemia gymnanthera*, *Smilax corbularia*, *Erigeron annuus*, and *Ficus tikoua*, were distributed in the highly similar habitats.

**Table 6:** Envift permutation function of the whole plant community and environmental factors

Factor	CCA1	CCA2	*r* ^2^	*P* (>*r*)
Stand density	0.90814	−0.41866	0.0893	0.259
Altitude	−0.87534	−0.4835	0.1585	0.117
Tree coverage	0.20138	−0.97951	0.2734	**0.014**
Bio 1	0.98241	0.18671	0.2144	0.074
Bio 2	0.9496	−0.31346	0.0655	0.408
Bio 3	0.39917	−0.91688	0.1192	0.174
Bio 4	0.40891	0.91258	0.1292	0.16
Bio 5	0.92058	0.39056	0.1343	0.146
Bio 6	0.99812	−0.06129	0.2137	0.059
Bio 7	0.57383	0.81898	0.0858	0.301
Bio 8	0.9664	0.25703	0.3993	**0.007**
Bio 9	0.90533	−0.42471	0.0587	0.487
Bio 10	0.90813	0.41869	0.1784	0.095
Bio 11	0.99999	−0.00333	0.273	**0.029**
Bio 12	−0.02045	−0.99979	0.0704	0.364
Bio 13	0.47377	−0.88065	0.1411	0.128
Bio 14	−0.88446	−0.46661	0.0505	0.505
Bio 15	0.93792	−0.34685	0.0964	0.252
Bio 16	0.41508	−0.90978	0.1336	0.128
Bio 17	−0.96026	−0.2791	0.0987	0.256
Bio 18	0.83912	−0.54395	0.0224	0.731
Bio 19	−0.76197	−0.64761	0.0663	0.404

## 4 Discussion

In this study, five SAD models were all accepted in both *P. orientalis* and *Pi. elliottii* shrub communities, indicating that different ecological processes coexisted and were not mutually exclusive. Most previous studies of SAD fitting focused on finding the best‐fitting model or tested a particular theory or model [38,39]. The fitting analysis of various models had great significance in assessing how different SAD properties changed across ecological gradients [26,27]. The different SAD models indicated that the two communities were homogeneous with a long history. The fitting results indicated that the SAD followed multiple rules, indicating that the community formation was affected by both random and deterministic processes [40,41]. The Zipf–Mandelbrot model was selected as the best-fitting model, which support hypotheses about underlying processes linking the requirements of various species with probabilities of encountering optimal growth conditions in the environment [40], indicating that deterministic processes may dominate. The comparison of two parameters indicated that the *P. orientalis* community had an unstable structure compared with the *Pi. elliottii* community, indicating that the *Pi. elliottii* understory community was easier to restore to the original state under disturbance. Compared with the *Pi. elliottii* community, the frequent immigration events led to a complex structure with a large number of rare species in *P. orientalis* community, indicating that the communities were in the stage of restoration succession, and niche overlap were common in these communities. We speculated that the reason was that the *P. orientalis* community had limited resources and space caused by the high resource requirements of the species, which was strongly resistant to drought, cold, diseases, and long lifespans [42]. The *Pi. elliottii* community can support more individuals, reducing the competitive interactions among species requiring a similar resource [43].

In this study, the understory communities of *P. orientalis* and *Pi. elliottii* across different scales (α and β) were integrated for the first time to fully understand the influence of pure *P. orientalis* and *Pi. elliottii* afforestation. The distribution pattern of two α indexes showed a unimodal curve along with altitude, consistent with the intermediate disturbance hypothesis and the niche-assembly hypothesis [44–46]. However, the irregular wave curve of α index could be attributed to the complex terrain of the study area, which was dominated by mountains and hills; these results were similar to those of other studies [47–49]. Environmental filtering and neutral processes often occur simultaneously and play different roles in different scale and ecosystem types [50,51]. Neutral processes may be dominant in species-rich communities, while environmental filtering may be dominant in communities with relatively few species (such as temperate forests) [52,53]. The linear regression between the β diversity and environmental/geographic distance indicated different construction mechanisms between *P. orientalis* and *Pi. elliottii* understory vegetation diversity. The understory vegetation of *Pi. elliottii* tended to be dominated by habitat filter, while that of *P. orientalis* tended to be dominated by both neutral processes and habitat filter. Species diffusion is one of the main stochastic processes, which is often measured by geographic distance [54]. The mantel test results indicated more frequent species diffusion events in *P. orientalis* communities. The partition of β diversity indicated that both communities were dominated by turnover processes, which were caused by environmental classification or spatial constraints. This suggested that maintaining habitat heterogeneity was essential in improving the understory vegetation diversity of pure *P. orientalis* and *Pi. elliottii* [55].

Previous studies showed that the turnover components often were dominant under natural conditions. Nestedness components appeared to be more evident in habitats with strong anthropogenic disturbance or habitats with considerable differences in species richness, such as island habitats [56–58]. In this study area, the ecological forest was protected and the forest management activities were strictly restricted, which might be one of the reasons for the spatial turnover dominance in β diversity.

## 5 Conclusions

This study reported the biodiversity patterns of the pure *P. orientalis* and *Pi. elliottii* understory vegetation communities in Central and Southern China for the first time. Five models were used to fit the SAD, which revealed its ecological process. MRT and ISA identified the domain factors and indicator species of each community. The analysis of β diversity indicated the different construction mechanisms between *P. orientalis* and *Pi. elliottii* understory vegetation diversity. Both communities were dominated by the turnover process, which reflected their formation history. Nevertheless, this study helped to understand the ecological process of diversity maintenance in the *P. orientalis* and *Pi. elliottii* understory vegetation communities, verifying the protective effect of ecological forest policy and thus providing theoretical guidance in the restoration process of the shrub diversity.

## Supplementary Material

Supplementary material
