# Supplementary material for: Understory vegetation diversity patterns of Platycladus orientalis and Pinus elliottii communities in Central and Southern China
Source: Open Life Sci. 2023 Dec 22;18(1):20220791. doi: 10.1515/biol-2022-0791 (PMC10752000; doi:10.1515/biol-2022-0791)
Supplement: Supplementary material [file biol-2022-0791-sm.pdf]

Supplementary material

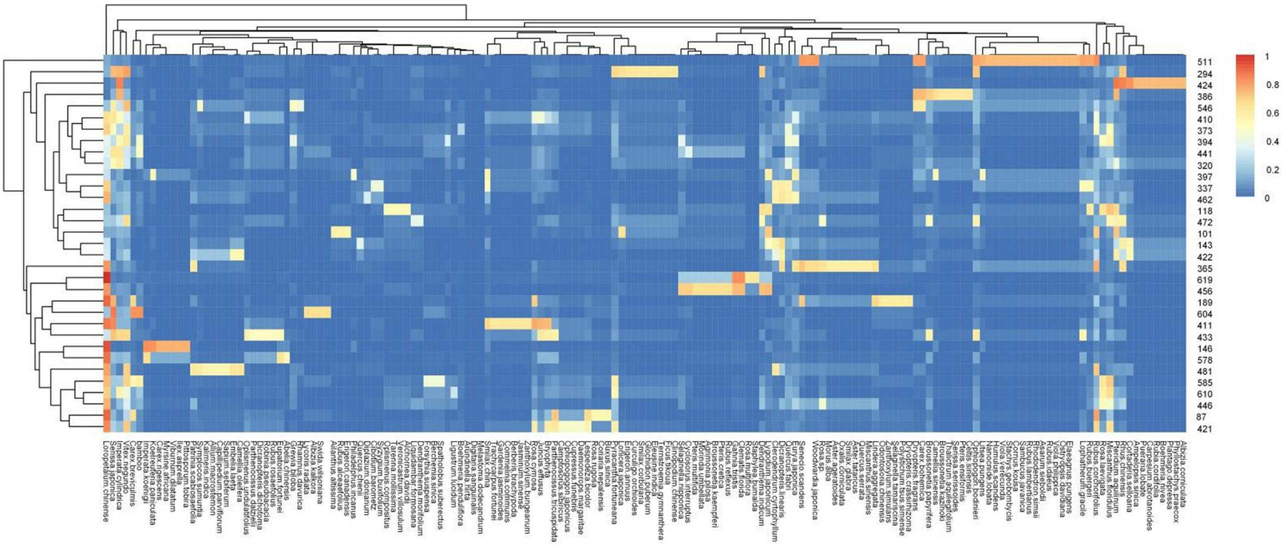

Figure S1: Heatmap of distribution probability of species in the plot.

Table S1: Nineteen bioclimatic variables

Code	Description
Bio1	Annual mean temperature
Bio2	Mean diurnal range [Mean of monthly (max temp−min temp)]
Bio3	Isothermality (Bio2/Bio7) (*100)
Bio4	Temperature seasonality (standard deviation × 100)
Bio5	Max temperature of the warmest month
Bio6	Min temperature of the coldest month
Bio7	Temperature annual range (Bio5–Bio6)
Bio8	Mean temperature of the wettest quarter
Bio9	Mean temperature of the driest quarter
Bio10	Mean temperature of warmest quarter
Bio11	Mean temperature of the coldest quarter
Bio12	Annual precipitation
Bio13	Precipitation of the wettest month
Bio14	Precipitation of the driest month
Bio15	Precipitation seasonality (coefficient of variation)
Bio16	Precipitation of the wettest quarter
Bio17	Precipitation of the driest quarter
Bio18	Precipitation of the warmest quarter
Bio19	Precipitation of the coldest quarter
